# Claims of Potential Expansion throughout the U.S. by Invasive Python Species Are Contradicted by Ecological Niche Models

**DOI:** 10.1371/journal.pone.0002931

**Published:** 2008-08-13

**Authors:** R. Alexander Pyron, Frank T. Burbrink, Timothy J. Guiher

**Affiliations:** 1 Department of Biology, The Graduate School and University Center, The City University of New York, New York, New York, United States of America; 2 Department of Biology, The College of Staten Island, The City University of New York, Staten Island, New York, United States of America; University of Zurich, Switzerland

## Abstract

**Background:**

Recent reports from the United States Geological Survey (USGS) suggested that invasive Burmese pythons in the Everglades may quickly spread into many parts of the U.S. due to putative climatic suitability. Additionally, projected trends of global warming were predicted to significantly increase suitable habitat and promote range expansion by these snakes. However, the ecological limitations of the Burmese python are not known and the possible effects of global warming on the potential expansion of the species are also unclear.

**Methodology/Principal Findings:**

Here we show that a predicted continental expansion is unlikely based on the ecology of the organism and the climate of the U.S. Our ecological niche models, which include variables representing climatic extremes as well as averages, indicate that the only suitable habitat in the U.S. for Burmese pythons presently occurs in southern Florida and in extreme southern Texas. Models based on the current distribution of the snake predict suitable habitat in essentially the only region in which the snakes are found in the U.S. Future climate models based on global warming forecasts actually indicate a significant contraction in suitable habitat for Burmese pythons in the U.S. as well as in their native range.

**Conclusions/Significance:**

The Burmese python is strongly limited to the small area of suitable environmental conditions in the United States it currently inhabits due to the ecological niche preferences of the snake. The ability of the Burmese python to expand further into the U.S. is severely limited by ecological constraints. Global warming is predicted to significantly reduce the area of suitable habitat worldwide, underscoring the potential negative effects of climate change for many species.

## Introduction

Ecological niche modeling is an effective method for predicting the ranges of species, identifying potential habitats, and assessing the likely impact of potential future climate change on species' distributions [1]. These methods use locality information on the presence of organisms within their range, combined with climatic data from those areas to generate models describing the ecological niche of the organism, estimate their current distribution and predict areas exhibiting the same or similar environmental space. Such methodologies have important applications in conservation biology for identifying regions where critically endangered species may be found, and areas where conservation actions may be taken to re-introduce populations. Likewise, ecological niche modeling may also be used to identify potential areas of suitable habitat for invasive species and highlight regions where preventative actions may be taken to limit the spread of alien organisms.

In a recent publication [2], United States Geological Survey (USGS) scientists generated a climate-based distribution model describing possible areas of suitable habitat for the exotic Burmese Python (*Python molurus*) in the United States. This species has been introduced into south Florida, primarily in the Everglades, as a result of escaped animals imported for the pet trade [3]. The distribution model predicts that a significant portion of the continental United States provides ecological conditions suitable for the snakes. A future climate model, based on global climate change scenarios, generated significantly expanded predictions that suggested the possible range for the Burmese python will extend into the states of Washington, Colorado, Illinois, Ohio and New York by the year 2100. The suggestion of an imminent giant python invasion has generated significant interest in the media, which in turn has spurred warnings of the dangers of large snakes spreading throughout the U.S. However, the methods employed by Rodda et al. [2] appear to deviate from the standard protocols used in ecological niche modeling, and may be significantly under-parameterized. Their model results in an apparent overprediction of the present-day distribution of the python in its native range, raising the possibility that the subsequent predictions for the United States are overly broad in representing potential areas of further colonization.

The Burmese or Indian Python (*Python molurus*) is native to the tropical rainforests and subtropical jungles of Burma, India, southern China, southeast Asia and parts of the Indonesian archipelago [4]. The biotic and abiotic factors which limit the distribution of this species in its native range are not entirely known, however, the abiotic factors may extend beyond the two variables used by Rodda et al. [2], mean monthly temperature and precipitation. Recently, the USGS has issued press releases [5] stating that the Burmese python could potentially expand throughout the eastern United States to the Great Plains and the Pacific coast. However, the models built by Rodda et al. [2] were not based on locality information for the pythons themselves, but on data gathered from weather stations stated to be within the present range of the Burmese python [2]. We wish to test the accuracy of these predictions using standard statistical techniques in niche modeling with locality data taken from the presence of pythons in their native range. Here, we use widely accepted modern methods of ecological niche modeling to generate both current range predictions and future predictions based on models of global climate change, and compare these to the results of Rodda et al. [2].

Statistical methods of ecological niche modeling, such as maximum entropy (implemented in the program MaxEnt [6] used here) take a set of presence localities and a set of climatic variables and fit a statistical distribution to those points which best describes the fundamental ecological niche with respect to the available climate data. Such methods perform particularly well with limited datasets of presence localities, and are commonly used to predict species ranges [7], [8]. We use actual presence locality data, as well as multiple climatic variables from the widely used Bioclim dataset [9] to generate models of the predicted current and potential range of the Burmese python. These models are based on significantly more information regarding the climatic variability encountered by the pythons than was examined by Rodda et al. [2]. The Bioclim dataset comprises not only measurements of mean temperature and rainfall, but extremes in those variables, as well as seasonal variability. This will more fully identify the climatic space which is suitable for habitation by the Burmese python, providing more accurate models for predicting areas of potential expansion. Additionally, we project these models onto the same future climate dataset used by Rodda et al. [2], representing a global warming scenario of increased greenhouse gases [10]. This allows us to examine the potential effects of global climate change on the predicted future distribution of the pythons.

## Results

**Figure 1:**
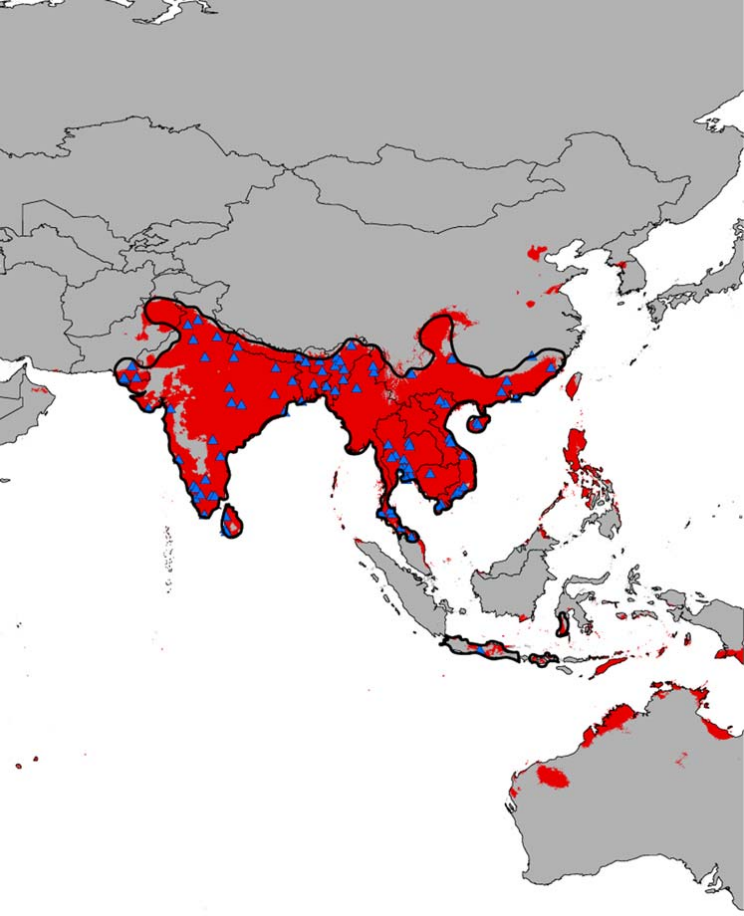
Current predicted range of the Burmese Python (*Python molurus*) in southeast Asia. Model was generated using the nineteen BIOCLIM variables. The solid black line indicates the approximate current distribution of the pythons [2]. The blue triangles indicate the presence localities. The minimum training presence threshold was used as a cutoff for suitability. Presence localities are detailed in Table S1.

The results from these models predict a current distribution which is remarkably concordant with the known range of the Burmese python (Fig. 1). Within the broad putative current range of the python, the snakes are predicted to be absent from portions of the Thar desert in Pakistan, as well as the highest peaks in the Western Ghats of India. The range of the python appears to be slightly underrepresented in southeastern China, as the locality record in the Jiangxi province of China was withheld by the MaxEnt algorithm as a training point used to assess the performance of the model. In other portions of the known range of the python, occurrences are correctly predicted on Java, the southern portion of Sulawesi, and the lesser Sunda islands. Some regions without known populations of the Burmese python are estimated to have suitable climate conditions, including the northern Philippines, the island of Timor and extreme northern Australia. These areas may result from imperfections in the predictions of our models. However, these regions may truly be suitable, with other climatic or biotic factors limiting the dispersal of the pythons to those areas. The predicted range of the Burmese python is very consistent with the known extent of its distribution.

**Figure 2:**
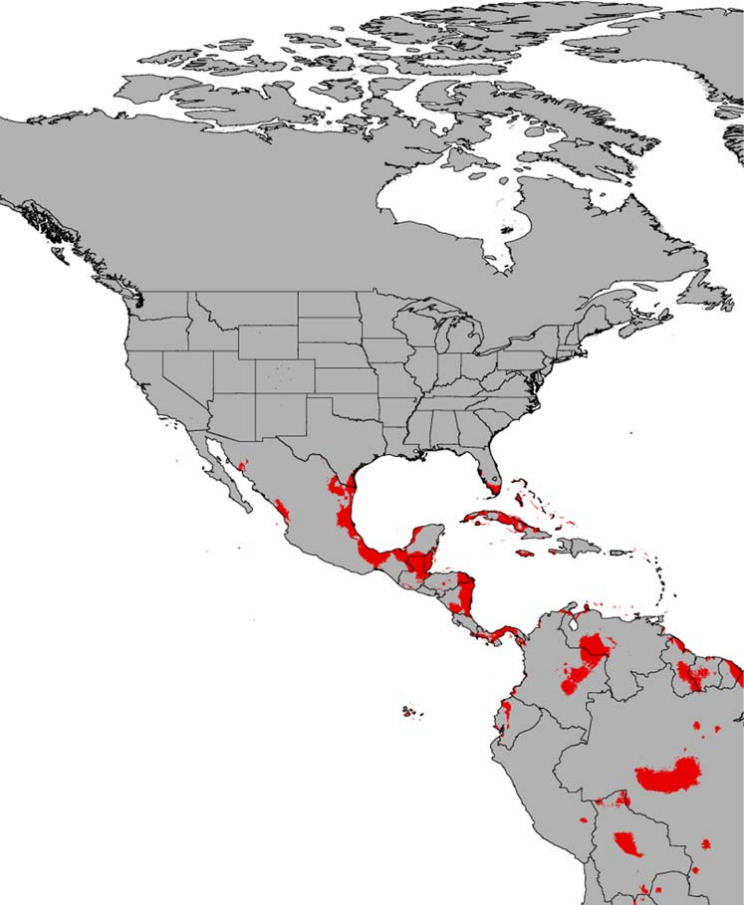
Current prediction of suitable habitat for *P. molurus* in the United States and adjacent Central America. No other areas in the U.S. were predicted as suitable outside of southern Peninsular Florida and southern Texas.

**Figure 3:**
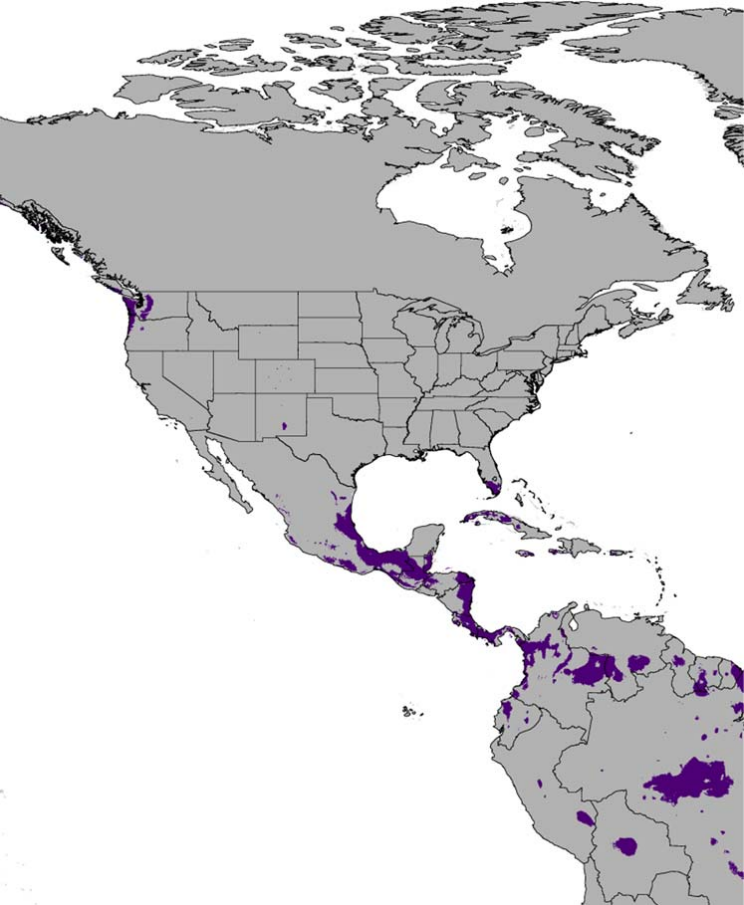
Future prediction of suitable habitat in the U.S. and surrounding areas for the Burmese python (*P. molurus*), based on projected global temperature increases generated from the CCM3 model.

**Figure 4:**
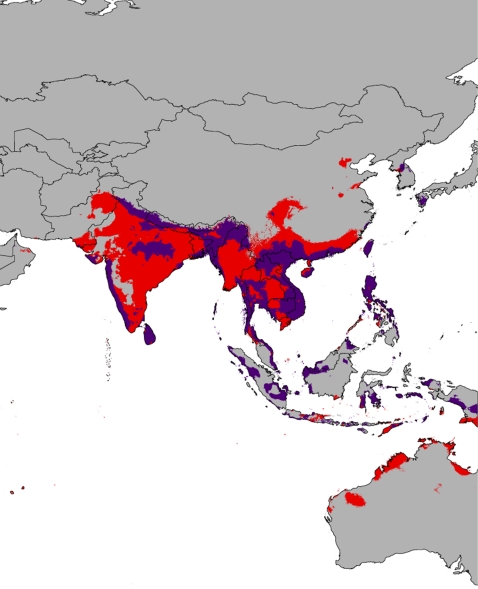
Predicted future suitable habitat for *Python molurus* in their native range under the CCM3 model of global climate change (purple), overlayed on their current predicted distribution (red), indicating the predicted contraction in suitable habitat.

In the United States, suitable habitat for the pythons is predicted to occur only in the southern portion of peninsular Florida and extreme southern Texas (Fig. 2). Other areas in the New World, such as many of the Caribbean islands are predicted as suitable, though no reports of colonization of those areas exist. Many of the Central and South American localities predicted as suitable habitat also possess their own boid snake fauna, such as *Boa constrictor* [4]. These native taxa could be threatened if these regions were to be colonized by Burmese pythons and competition and displacement occurred. The future predictions of potential distribution based on forecasts of global climate change from the CCM3 model [11] predict a worldwide contraction in the suitable habitat available for the python (Fig. 3, 4). Both within the native range of the snakes as well as in the United States, the areas of potential occupancy are diminished. Within the U.S., the available niche disappears from southern Texas and shrinks in southern Florida. Somewhat unexpectedly, a large portion of suitable environmental space is predicted to become available in the northwestern United States under the models of potential global climate change (Fig. 3).

## Discussion

Our results indicate that the models generated by Rodda et al. [2] are excessively broad and do not represent an accurate picture of the potential current or future extent of the Burmese python in the United States. Our predicted occurrence of the pythons in their native habitat largely coincides with their current range (Fig. 1) The models generated using the 19 climatic variables indicate a very restricted area of suitable environmental conditions for the Burmese python in the United States, confined primarily to the extreme southern portion of peninsular Florida in the Everglades, and extreme southern Texas (Fig. 2). Records of pythons exist from several localities outside of this prediction [2]–[4], but these appear to be isolated releases, and do not represent established populations. The only breeding populations of Burmese pythons in the U.S. are found in the Everglades National Park and Big Cypress National Preserve in extreme southern Florida [2], [5]. One of the most striking aspects of our results is that the primary area of predicted suitability in the United States, the Everglades, is the only place that the pythons have ever been found to have established populations. This concordance is particularly persuasive, as no information regarding the presence of the pythons in the U.S. was used to generate the model. The pythons are primarily predicted to occur in the U.S. in the only place that they have been found to occur, despite the widespread presence and availability of these snakes as pets throughout the country.

The Everglades form an ecosystem which is unique in the continental United States, a subtropical marshland that may be quite similar to the Burmese python's preferred native environment. However, the extent of this ecosystem is limited to extreme southern Florida; the rest of the peninsula is composed of xeric highlands and mesic subtropical lowlands. The proposed expansion of the python into the continental United States would require an expansion of the actual tropical marshland habitat comprising most of the Everglades, not simply the presence of similar temperature and precipitation conditions. The future predicted distribution (Fig. 3, 4) indicates that the area of suitable habitat for the Burmese python would contract following a global temperature increase. In contrast to the forecast of Rodda et al. [2], the only large area of suitable habitat predicted in the continental United States by the CCM3 model occurs within the confines of Everglades National Park. Additionally, other areas are predicted to contract significantly or disappear all together (Fig. 3, 4).

In comparison to our results using standard niche modeling techniques, the primary problem with the model and predictions used by Rodda et al. [2] is that only two climatic variables (mean monthly temperature and mean monthly rainfall) were used, and that the model lacked true presence records for the Burmese python. The authors gathered data from weather stations which were said to lie within the range of the pythons, then generated a climatic envelope putatively describing the range of temperature and rainfall which exist within the native habitat of the pythons. They then projected this model onto climatic data for the United States, which identified the areas exhibiting similar conditions in terms of average rainfall and temperature. However, modeling species' distributions as a function of two components that reflect the average rather than the limits of the fundamental niche will lead to extremely wide inferences, as appears to be the case in Rodda et al. [2]. Figure 3 of Rodda et al. [2] illustrates the prediction for the Burmese python in its current native range, and includes vast areas where the python has never been recorded in modern times, including Afghanistan, Borneo and the Arabian peninsula. The fact that their model makes such inaccurate predictions of the current distribution in the native range of this species questions the use of their model in identifying potential areas of future invasion. Modeling the ecological niche of organisms solely as a function of mean temperature and precipitation omits the critical interactions between these variables that define the fundamental and realized niche which organisms inhabit [12], [13]. If species were able to expand into environments simply based on mean precipitation and temperature conditions similar to those in their native range, the continental United States should be overrun by the northward expansion of subtropical species such as the Boa Constrictor, found only 145 km south of the Texas border.

We do not intend to downplay the critical problems that invasive wildlife pose for native ecosystems, or the possible impact that global climate change may have on the environment. The establishment of the Burmese python in the Everglades poses a serious risk to the balance of the park's ecosystem. However, the results from the ecological niche modeling suggest that the possibility for the expansion of the species into the rest of the United States is vastly overstated, and citizens throughout most of North America should have no fear of giant snakes invading their neighborhoods. Based on our results, the methods and predictions of Rodda et al. [2] seem particularly imprecise and do not appear to represent an accurate picture of the threat posed by the Burmese pythons. The alarmist claims made by the USGS could potentially hamper scientific discourse and inquiry into the problem, especially with regard to policy-making. Here, we have demonstrated that the suitable niche for the Burmese Python in the United States is minimal, based on available data regarding the current distribution of the snakes. Additionally, global warming is projected to have a negative effect on the species' continued survival. Climate change models indicate that the effects of global warming will result in a drastic decrease in the suitable habitat for the Burmese python, both in the United States and its native range. These serious problems do not benefit from any potential exaggeration of possible threats to the public and the legislature.

In summary, we find that statistically generated ecological niche models based on presence localities for the Burmese python and extensive climatic datasets predict the current distribution of the snakes with a high degree of accuracy. This suggests that these models will be particularly useful in identifying other areas of extralimital habitat. Regarding areas of putative suitability and potential expansion within the United States, we find, remarkably, that the area in which the snakes are known to have colonized (south Florida) is essentially the only region where the climatic conditions are suitable for the pythons. Almost no potential for further continental expansion is predicted based on the results from the ecological niche models. Additionally, we find that under projected models of global climate change the worldwide suitable habitat decreases significantly. This is in contrast to the results of Rodda et al. [2]. Based on the results of our analyses, we find that global climate change has the potential to greatly reduce the available habitat for the pythons, especially in their native range (Fig. 4). This pattern is likely not unique to the Burmese python and may potentially apply to many species, not only snakes.

## Materials and Methods

We modeled the potential distribution of *Python molurus* worldwide in both the current environment and a potential future environment using the maximum entropy method of ecological niche modeling (MaxEnt ver. 3.2.1 [6]). MaxEnt has been shown to be a robust method for presence only datasets [7] and is quickly becoming one of the more widely used methods of ecological niche modeling [8]. We used the WorldClim dataset [9], which comprises nineteen temperature and precipitation variables (BioClim) interpolated across the globe at up to 30 second (1 km^2^) resolution. In order to train the models, we collected ninety data points throughout the native range of *P. molurus*. These were georeferenced from museum voucher specimens and occurrences reported in the literature or from wildlife agencies (Table S1). Presence localities were used to train the ecological niche model for *P. molurus* on the nineteen BioClim variables across the world at a 2.5 min (21.6 km^2^) spatial resolution and produce a predicted present-day distribution (Fig. 1). In order to estimate the effect of global climate change on the potential distribution of *P. molurus*, we projected our model onto the climatic predictions generated by the CCM3 community circulation model [10] representing temperature increases following hypothesized greenhouse gas increases. This is the same future climate model utilized by Rodda et al. [2] We applied the default regularization parameter (1.0), and allowed the algorithm to choose the most appropriate “feature” type based on sample size by utilizing the auto-feature option [6]. The “fade by clamping” feature was applied to the future predictions to reduce the effect of projecting onto environmental conditions not present in the training dataset. A threshold of 0.160 was applied to the predictions based on the minimum training presence [11]. This minimized both the training and test omission rates without resulting in an overly general model. We assessed model performance by allowing the model to randomly designate ten percent of the samples as test data in order to calculate the area under the receiver operating characteristic curve (AUC/ROC [7]). The model performed significantly better than random with both AUC values greater than 0.9 (training AUC  =  0.988, test AUC  =  0.978).

## Supporting Information

Table S1 Presence Localities. The presence localities used to generate ecological niche models for Python molurus.
(0.19 MB DOC)
