# Supplementary material for: Claims of Potential Expansion throughout the U.S. by Invasive Python Species Are Contradicted by Ecological Niche Models
Source: PLoS One. 2008 Aug 13;3(8):e2931. doi: 10.1371/journal.pone.0002931 (PMC2490718; doi:10.1371/journal.pone.0002931)
Supplement: Table S1 — Presence Localities. The presence localities used to generate ecological niche models for Python molurus. (0.19 MB DOC) [file pone.0002931.s001.doc]

Table 1: Museum number, reference, locality and Lat/Long for *P. molurus*  presence records.

| Ref./Museum No. | Country | Province | Locality | Lat | Long |
| --- | --- | --- | --- | --- | --- |
| [1] | Bangladesh | Dhaka | Bhawal National Park | 24.01666700 | 90.33333000 |
| FMNH 259166 | Cambodia | Siem Reap | Siem Reap District | 13.46905400 | 104.04087400 |
| [2] | China | Fujian |  | 25.93976800 | 118.28360100 |
| [3] | China | Guangdong | Shimentai Nature Reserve | 24.36666700 | 113.08333200 |
| [4] | China | Guangdong | Dinghushan National Natural Reserve | 23.13833300 | 112.43666700 |
| [2] | China | Guizhou |  | 26.92268000 | 106.59409000 |
| MVZ 23614 | China | Hainan | Hainan Island | 19.50000000 | 109.55000000 |
| MVZ 23614 | China | Hainan |  | 19.19293400 | 109.74578100 |
| [5] | China | Hong Kong |  | 22.28330000 | 114.15000000 |
| AMNH R-35231 | China | Yunnan |  | 25.19783200 | 101.86166000 |
| [6] | India | Andra Pradesh | Nallamalai Hills | 15.47500000 | 79.33500000 |
| [7], Park Wildlife Info | India | Andra Pradesh | Kasu Brahmananda Reddy NP | 17.42147900 | 78.42086100 |
| [8] | India | Arunachal Pradesh | Eaglenest Wildlife Sanctuary | 27.10000000 | 93.00000000 |
| [9] | India | Arunachal Pradesh | Mouling National Park | 28.54083300 | 94.76555600 |
| ROM 17070 | India | Assam |  | 26.13684700 | 92.85179000 |
| [10] | India | Assam | Manas Wildlife Sanctuary | 26.72500000 | 91.03060000 |
| [11] | India | Assam | Kaziranga National Park | 26.66670000 | 93.41670000 |
| [7] | India | Bihar |  | 25.92507000 | 85.85539900 |
| CAS-SU (Rep) 13678 | India | Chhattisgarh | Tilda | 21.55377000 | 81.79886600 |
| [12] | India | Gujarat | Vansda National Park | 21.10500000 | 73.43000000 |
| [7], Park Wildlife Info | India | Gujarat | Gir National Park | 21.13563700 | 70.79658500 |
| [7] | India | Haryana |  | 29.24541000 | 76.11967200 |
| [7] | India | Himachal Pradesh |  | 31.57194400 | 76.68027100 |
| [7] | India | Jharkand |  | 22.79354000 | 85.68376600 |
| [13] | India | Karnataka | Nagarhole National Park | 12.02701800 | 76.14006000 |
| [13] | India | Karnataka | Bandipur National Park | 11.70779100 | 76.45523100 |
| [13] | India | Karnataka | Anshi National Park | 15.07237300 | 74.39598100 |
| [7], Park Wildlife Info | India | Karnataka | Bannerghatta National Park | 12.76266800 | 77.56343400 |
| [7] | India | Kerala |  | 10.52881900 | 76.35232000 |
| [7], Park Wildlife Info | India | Madhya Pradesh | Bandhavgarh National Park | 23.61267100 | 80.38696300 |
| [7], Park Wildlife Info | India | Madhya Pradesh | Kanha National Park | 21.80774100 | 80.62179600 |
| [7], Park Wildlife Info | India | Manipur | Keibul Lamjao National Park | 24.54575900 | 93.81191800 |
| [7] | India | Meghalaya |  | 25.53401600 | 91.33066600 |
| [7] | India | Mizoram |  | 23.23980900 | 92.84655700 |
| [7] | India | Nagaland |  | 25.72602700 | 93.85665100 |
| [7], Park Wildlife Info | India | Orissa | Bhitarkanika National Park | 20.67781000 | 86.90365800 |
| [7] | India | Punjab |  | 30.98348800 | 75.43349400 |
| [14] | India | Rajasthan | Keoladeo National Park | 27.15890000 | 77.50860000 |
| [7], Park Wildlife Info | India | Sikkim | Khangchendzonga National Park | 27.17555800 | 88.52727500 |
| CAS 12250 | India | Tamil Nadu |  | 10.81015600 | 78.30068800 |
| CAS-SU (Rep) 12250 | India | Tamil Nadu | Trichinopoly | 10.80093300 | 78.68133500 |
| [15] | India | Tamil Nadu | Kalakad-Mundanthurai Tiger Reserve | 8.58333300 | 77.41667000 |
| [16] | India | Tamil Nadu | Coimbatore | 11.00520000 | 76.97071100 |
| [7] | India | Tripura |  | 23.74345000 | 91.80971400 |
| [7] | India | Uttar Pradesh |  | 27.13781700 | 80.90234700 |
| [7] | India | Uttarakhand |  | 29.58572500 | 78.93577100 |
| CU 11412 | India | West Bengal |  | 24.36865300 | 87.81354800 |
| [17] | India | West Bengal | Gorumara National Park | 26.61214400 | 89.37309300 |
| [17] | India | West Bengal | Sundarbans National Park | 21.94860400 | 88.88893500 |
| USNM 7516 | Indonesia | Java |  | -7.32724200 | 109.90127600 |
| [18] | Macau | Coloane | Estrada Choc-van | 22.12319400 | 113.56117900 |
| [18] | Macau | Taipa | Hovione Chemical Plant | 22.15876500 | 113.56479200 |
| USNM 122197 | Myanmar | Kachin | Myitkyina | 25.38300000 | 97.40000000 |
| USNM 122197 | Myanmar | Kachin |  | 26.09241400 | 97.30942500 |
| Park Wildlife Info | Myanmar | Sagaing | Kanbalu, Kyatthin Wildife Sanctuary | 23.50000000 | 95.40000000 |
| [19] | Nepal | Bheri | Bardia National Park | 28.41797500 | 81.35925300 |
| [20] | Pakistan | Sindh | Mithi, Thar Desert | 24.76670000 | 69.38330000 |
| CAS 99811 | Pakistan | Sujawal |  | 24.60320000 | 68.07950000 |
| TCWC 23870 | Pakistan | Sindh |  | 26.11206200 | 68.88789800 |
| AMNH R-102281 | Pakistan | Sindh | Tatta | 24.75340000 | 67.92210000 |
| AMNH R-85533 | Sri Lanka | Western | Colombo | 6.93114800 | 79.87963000 |
| Park Wildlife Info | Sri Lanka | North Western | Wilpattu National Park | 8.39213900 | 79.97570000 |
| USNM 267819 | Sri Lanka | Western | Kalutara | 6.59349200 | 79.99990500 |
| [21] | Thailand | Chachoengsao | Khao Ang Rue Nai | 13.23333300 | 101.75000000 |
| [22] | Thailand | Chon Buri |  | 13.17935300 | 101.12816900 |
| [21] | Thailand | Loei | Phu Luang | 17.12155400 | 101.54667800 |
| [22] | Thailand | Lopburi |  | 15.10763000 | 100.90986600 |
| [21] | Thailand | Nakhon Nayok | Ongkarak | 14.12051100 | 101.02571400 |
| [21] | Thailand | Nakhon Sawan |  | 15.62140000 | 99.95999300 |
| [21] | Thailand | Nakhon Si Thammarat | Khao Luang | 8.72493300 | 99.59381100 |
| [21] | Thailand | Narathiwat | Waeng | 5.90833300 | 101.86666600 |
| [21] | Thailand | Pathum Thani | Rang Sit | 13.99020900 | 100.60234000 |
| [21] | Thailand | Phang Nga | Khao Lak | 8.72046600 | 98.26215000 |
| [21] | Thailand | Phetchabun | Nam Nao | 16.69703800 | 101.63246200 |
| [21] | Thailand | Prachin Buri | Prachantakham | 14.05918000 | 101.52629900 |
| [22] | Thailand | Rayong |  | 12.74184300 | 101.25861900 |
| [21] | Thailand | Songkhla |  | 7.11439800 | 100.57989100 |
| [21] | Thailand | Surat Thani |  | 9.13459900 | 99.33394600 |
| [21] | Thailand | Tak |  | 16.87483200 | 99.11600200 |
| [21] | Thailand | Uthai Thani |  | 15.34465600 | 99.49430100 |
| ROM 30946 | Vietnam | Bac Thai |  | 21.68980600 | 105.84320000 |
| FMNH 11519 | Vietnam | Cochinchina |  | 11.00000000 | 107.00000000 |
| [23] | Vietnam | Dong Nai | Cat Tien National Park | 11.41541800 | 107.39135700 |
| USNM 292077 | Vietnam | Kien Giang |  | 9.90423700 | 105.31686500 |
| [24] | Vietnam | Kien Giang | U Minh Thuon National Park | 9.58333300 | 105.08333000 |
| USNM 95101 | Vietnam | Lam Dong |  | 11.88723700 | 108.01554500 |
| [25] | Vietnam | Quang Binh | Phong Nha-Ke Bang NP | 17.58467300 | 106.28129000 |
| [26] | Vietnam | Quang Binh | Truong Son | 17.11666700 | 106.50000000 |
| USNM 163823 | Vietnam | Quang Nam |  | 15.59456800 | 107.96576400 |
| ROM 30795 | Vietnam | Tuyen Quang |  | 22.06427200 | 105.20504000 |

Museum abbreviations are as follows: USNM, United States National Museum; FMNH, Field Museum of Natural History; AMNH, American Museum of Natural History; MVZ, Museum of Vertebrate Zoology; ROM, Royal Ontario Museum; CAS, California Academy of Sciences; CU, Cornell University Museum of Vertebrates; TCWC, Texas Cooperative Wildlife Collection. ‘Park Wildlife Info’ refers to informal fauna checklists provided by parks or associated agencies.

1 Kabir DS, Ahmed AZ (2005) Wildlife Biodiversity in Bhawal National Park: Management Techniques and Drawbacks of Wildlife Management and Nature Conservation. Our Nature 3: 83-90.

2 Zhao EM, Adler K (1993) Herpetology of China. Oxford, OH: Society for the Study of Amphibians and Reptiles. pp 522.

3 Xu SSW, Jim CY (2003) Using upland forest in Shimentai Nature Reserve, China. Geog. Rev. 93: 309-327.

4 PRC State Environmental Protection Administration (1999) Report on the State of the Environment in China, Bio-Diversity Report. Available: <http://www.zhb.gov.cn/english/SOE/soechina1999/biod/biod.htm>. Accessed 31 March 2008

5 Karsen SJ, Michael W, Lau WN, Bogadek A (1998) Hong Kong Amphibians and Reptiles. Hong Kong: Hong Kong Urban Council. ed. 2, pp. 186.

6 Rao TR, Ghate HV, Sudhakar M, Javed MSM, Siva Rama Krishna I (2005) Herpetofauna of Nallamalai Hills with Eleven New Records from the Region Including Ten New Records for Andhra Pradesh. Zoos’ Print J. 20: 1737-1740.

7 Whitaker R, Captain A (2004) Snakes of India: The Field Guide. Chennai, IN: Draco Books. pp. 481.

8 Athreya R (2006) Eaglenest Biodiversity Project − I (2003 – 2006): A report submitted to the Forest Department of the Government of Arunachal Pradesh, India, and the Rufford-Maurice-Laing Foundation (UK). Pune, IN: Kaati Trust. Available: <http://www.ncra.tifr.res.in/~rathreya/Eaglenest/ebp1reportW.pdf>. Accessed 31 March 2008.

9 Chatterjee S, Abhinandan S, Dutta P, Ghosh D, Pangging G et al. (2006) Biodiversity Significance of Northeast India. Background Paper No. 13. New Delhi: WWF-India. Available: <http://mdoner.gov.in/writereaddata/sublink3images/40.pdf>. Accessed 31 March 2008

10 Rahmani A, Narayan G, Rosalind L (1989) Threat to India's Manas Tiger Reserve. New Delhi: Ministry of Environment and Forests.

11 Spillett J (1966) A report on wild life surveys in North India and southern Nepal: the Kaziranga Wild Life Sanctuary, Assam. J. Bombay Nat. Hist. Soc. 63: 494-533.

12 Vyas R (2004) Herpetofauna of Vansda National Park, Gujarat. Zoos’ Print J. 19: 1512-1514.

13 Western Ghats Biodiversity Information System. National Parks of the Western Ghats. Bangalore, IN: Center for Ecological Science. Available: <http://ces.iisc.ernet.in/biodiversity/documents/national.htm>. Accessed 31 March 2008.

14 K. Bhatt (1991) The Diet Activity Pattern of Indian Python (*Python molurus molurus* Linn) at Keoladeo National Park and Some Factors Influencing It. Thesis, Saurashtra University, Gujarat, India.

15 Johnsingh JT (2001) The Kalakad-Mundanthurai Tiger Reserve: A Global Heritage of Biological Diversity. Current Sci. 80: 378-388.

16 Kannan P, Gokula V, Umapathy G, Venkatraman C (1998) Occurrence of the Indian Python *(Python molurus*) near Coimbatore, Tamil Nadu. Cobra 31: 19.

17 Alfred JRB, Sanyal AK, Tiwari S, Mitra S (2005) Status of Biodiversity of West Bengal. Kolkata: West Bengal Department of Environment. Available: <http://www.enviswb.gov.in/ENV/downloads/DL/BDFINALTECH/wb.htm>. Accessed 31 March 2008.

18 Easton ER, Leung-Va (1993) An Annotated List of the Reptiles and Amphibians of Macau, Southeast Asia. Herp. Rev. 24: 158-160.

19 Basnet K (1995) Biodiversity Inventory of Royal Bardia National Park, Bardia. Kathmandu: WWF Nepal Program and Department of National Parks and Wildlife Conservation.

20 Isani GB (2000) Status Paper on Situation of Arid Zones of Sindh. Karachi, Pakistan: IUCN – The World Conservation Union Sindh Programme. pp. 32.

21 Nabhitabhata J, Chan–ard T (2005) Status of Mammals, Reptiles and Amphibians in Thailand. Bangkok: Office of Natural Resources and Environmental Policy and Planning. Available: <http://chm-thai.onep.go.th/publication/doc/onep14/onep_v14_05-rep.pdf>. Accessed 31 March 2008.

22 Humphrey SR, Bain JR (1990) Endangered animals of Thailand. Gainesville, FL: Sandhill Crane Press. pp. 480.

23 Phan Dong Vat (1992) Red Data Book of Vietnam. Hanoi: Science and Technics Publishing House. vol. 1. pp. 396.

24 Stuart BL (2004) The harvest and trade of reptiles at U Minh Thuong National Park, southern Viet Nam. TRAFFIC Bull. 20: 25-34.

25 Ziegler **T, Hendrix R, Vu Ngoc Thanh, Vogt M, Forster B et al. (2007)** The diversity of a snake community in a karst forest ecosystem in the central Truong Son, Vietnam, with an identification key. Zootaxa 1493: 1-40.

26 T. Ziegler, Ohler A, Thanh VN, Quyet LK, Thuan NX et al. (2006) Review of the Amphibian and Reptile Diversity of Phong Nha – Ke Bang National Park and Adjacent Areas, central Truong Son, Vietnam. In: Vences M, Köhler J, Ziegler T, Böhme W, editors. Herpetologia Bonnensis II. Proceedings of the 13th Congress of the Societas Europaea Herpetologica. pp. 247-262.
